# Reply to: The challenge of cardiac dose constraint adaptation to hypofractionated breast radiotherapy in clinical practice

**DOI:** 10.1007/s00066-021-01775-4

**Published:** 2021-04-23

**Authors:** Marc D. Piroth, David Krug, Gerd Fastner, Felix Sedlmayer, Wilfried Budach, Breast Cancer Expert Panel of the German Society of Radiation Oncology (DEGRO)

**Affiliations:** 1 Department of Radiation Oncology, Helios University Hospital Wuppertal, Witten/Herdecke University, Heusnerstraße 40, 42283 Wuppertal, Germany; 2 Department of Radiation Oncology, University Hospital Schleswig-Holstein, Kiel, Germany; 3 Department of Radiation Oncology, Paracelsus Medical University Hospital, Salzburg, Austria; 4 Department of Radiation Oncology, Heinrich-Heine-University Hospital, Düsseldorf, Germany

We thank Dr. Loap and Dr. Kirova for their thoughtful comments. In principle, we can support the comments. We agree that with ultra-hypofractionation, such as the regimens tested in the FAST and FAST-Forward trials [1, 2], caution should be exercised with regard to cardiac constraints. The recommended dose constraints [3] cannot simply be adopted for ultra-hypofractionation in the case of breast cancer radiotherapy. The FAST-Forward trial protocol recommended to keep the volume of the heart receiving 7 Gy (Gray) and 1.5 Gy to less than 5% and less than 30%, respectively. So far, only 6‑year data are available for the FAST-Forward trial with regard to cardiac toxicity. This is too early to be able to make reliable recommendations regarding cardiac side effects.

For moderate hypofractionation with 15–16 fractions of 2.6–2.7 Gy, we refer to the detailed calculations and conclusions of Appelt et al. [4]. The authors showed that moderate hypofractionation results in a lower radiogenic burden on heart structures as compared to conventional fractionation. In our opinion the calculations previously published by Loap et al. [5] are in line with the results from Appelt et al. Also, long-term data from trials with moderate hypofractionation [6, 7] demonstrate that no increased cardiac toxicity is to be expected [8].

**Fig. 1:**
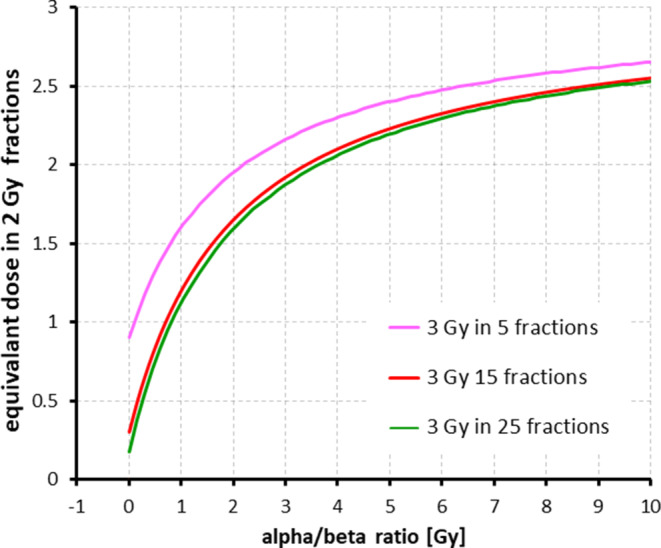
Equivalent dose on 2-Gy fractions (EQD2) for different fractionation regimens depending on several alpha/beta values assuming a mean herat dose of 3 Gy

The need for caution with regard to ultra-hypofractionation and adoption of cardiac constraints is illustrated in Fig. 1. For different fractionation regimens and assuming a mean heart dose of 3 Gy, the alpha/beta values are plotted against equivalent dose in 2‑Gy fractions (EQD2). It is shown that the graphs are almost congruent for normofractionation and moderate hypofractionation, corresponding to a comparable biological equivalent mean heart dose. By contrast, the graph representing the ultra-hypofractionation shows higher equivalent mean heart doses for all alpha/beta values.

In summary, if adjusted to EQD2 within the alpha/beta model, we consider it well justifiable to recommend our published cardiac dose constraints for moderately hypofractionated regimens. However, especially if ultra-hypofractionation is used, due to an unknown degree of biological uncertainty and due to the short follow-up, further scientific work is essential to draw definite conclusions.
